# Is the Karyotype of Neotropical Boid Snakes Really Conserved? Cytotaxonomy, Chromosomal Rearrangements and Karyotype Organization in the Boidae Family

**DOI:** 10.1371/journal.pone.0160274

**Published:** 2016-08-05

**Authors:** Patrik F. Viana, Leila B. Ribeiro, George Myller Souza, Hipócrates de Menezes Chalkidis, Maria Claudia Gross, Eliana Feldberg

**Affiliations:** 1 Instituto Nacional de Pesquisas da Amazônia, Coordenação de Biodiversidade, Av. André Araujo 2936, Petrópolis, CEP: 69067-375 Manaus, AM, Brazil; 2 Universidade Federal do Amazonas, Instituto de Ciências Biológicas, Rua General Rodrigo Otávio Num. 3000, Mini-Campus Coroado, CEP: 66077070 Manaus, AM, Brazil; 3 Faculdades Integradas do Tapajós, Laboratório de Pesquisas Zoológicas, Santarém, PA, Brazil; 4 Criadouro Comercial Jiboias Brasil, Belo Horizonte, MG, Brazil; Institute of Cotton Research of Chinese Academy of Agricultural Sciences, CHINA

## Abstract

Boids are primitive snakes from a basal lineage that is widely distributed in Neotropical region. Many of these species are both morphologically and biogeographically divergent, and the relationship among some species remains uncertain even with evolutionary and phylogenetic studies being proposed for the group. For a better understanding of the evolutionary relationship between these snakes, we cytogenetically analysed 7 species and 3 subspecies of Neotropical snakes from the Boidae family using different chromosomal markers. The karyotypes of *Boa constrictor occidentalis*, *Corallus hortulanus*, *Eunectes notaeus*, *Epicrates cenchria* and *Epicrates assisi* are presented here for the first time with the redescriptions of the karyotypes of *Boa constrictor constrictor*, *B*. *c*. *amarali*, *Eunectes murinus* and *Epicrates crassus*. The three subspecies of *Boa*, two species of *Eunectes* and three species of *Epicrates* exhibit 2n = 36 chromosomes. In contrast, *C*. *hortulanus* presented a totally different karyotype composition for the Boidae family, showing 2n = 40 chromosomes with a greater number of macrochromosomes. Furthermore, chromosomal mapping of telomeric sequences revealed the presence of interstitial telomeric sites (ITSs) on many chromosomes in addition to the terminal markings on all chromosomes of all taxa analysed, with the exception of *E*. *notaeus*. Thus, we demonstrate that the karyotypes of these snakes are not as highly conserved as previously thought. Moreover, we provide an overview of the current cytotaxonomy of the group.

## Introduction

Cytotaxonomy plays a key role in elucidating the taxonomy and chromosomal evolution of snakes [1–5], particularly when morphological characteristics are insufficient for the resolution of taxonomic problems.

Cytotaxonomic studies are important in the Neotropical region where many taxa have high cryptic diversity [6, 7] because they enable the efficient detection of structural changes in the genomes of these animals through the use of chromosomal markers. These karyotypic variations were well documented in 2 subspecies of true coral snakes of the genus *Micrurus* in Costa Rica [8], which were later elevated to the species category due to these variations in different populations.

The family Boidae is an excellent model for studies of this type because the family members are morphologically and biogeographically divergent along their wide distribution in the Neotropical region [9, 10]. The basal species of the family (*Boa constrictor*) comprise 9 subspecies that differ in many characteristics, such as body size, the distribution patterns of spots on the skin, behaviour, and diet [9, 11]. Recently, Hynková et al. [9] demonstrated the existence of distinct groups among the subspecies of *B*. *constrictor* using molecular markers, with the formation of 1 clade for Central America and another clade for the subspecies of South America. This result suggests that the subspecies *B*. *constrictor imperator* should be assigned the elevated rank of full species.

The arboreal boas of the genus *Corallus* currently comprise 9 valid species. Similar to the species and subspecies of *B*. *constrictor*, they present many morphological variations along their wide distribution in the Neotropical region [12, 13]. Three species can be found in both Central America and South America (*Corallus blombergi*, *Corallus annulatus* and *Corallus ruschenbergerii*). Additionally, two species are endemic to the West Indies (*Corallus cookii* and *Corallus grenadensis*), and 4 species can be found in South America, specifically in Brazil (*Corallus cropanii*, *Corallus caninus*, *Corallus batesii* and *Corallus hortulanus*).

The genus *Eunectes* comprises 4 species commonly known as anacondas: *Eunectes notaeus* and *Eunectes deschauenseei*, which are considered yellow anacondas, and *Eunectes murinus* and *Eunectes beniensis*, which are considered green anacondas [14,15]. These species are predominantly aquatic snakes and are widely distributed in South America [16, 17].

The genus *Epicrates* (rainbow boas) comprises 5 species that are distributed in South America [15, 18]. Until recently, only 1 species of this genus had been observed in Brazil (*Epicrates cenchria*); this species is widely distributed in different biomes and is subdivided into 9 subspecies. However, in a taxonomic review based on morphological characteristics, these subspecies were reorganized into 5 full species, the following 4 of which occurred in Brazil: Caatinga Rainbow Boa (*Epicrates assisi*), Cerrado Rainbow Boa (*Epicrates crassus*), Amazon Rainbow Boa (*Epicrates cenchria*) and Northern Rainbow Boa (*Epicrates maurus*) [15, 18,19].

These snakes have a relatively stable karyotype with highly conserved chromosomes [1, 2, 20]. They have a diploid chromosomal complement (2n = 36 and 16M + 20mi), which is also present in many species of the suborder Serpentes in derived lineages of the Colubroidea superfamily, such as Dipsadidae and Viperidae [21, 22].

In the Boidae family, variation of 2n = 36 to 2n = 44 chromosomes exists. More complete chromosomal data are available only for *B*. *constrictor*, including rDNA data obtained by staining with silver nitrate (AgNO_3_) for the subspecies *B*. *constrictor amarali* [23] and data obtained by chromosomal mapping with fluorescence *in situ* hybridization (FISH) to show homomorphic sex chromosomes [24]; however, the latter study did not indicate the *B*. *constrictor* subspecies. Within the superfamily Booidea, only repetitive DNA sequences from the family Sanziniidae (*Sanzinia madagascariensis*) have been mapped by FISH [25]; this mapping revealed the presence of telomeric motifs only in the terminal regions of chromosomes and a lack of interstitial telomeric sites (ITSs), which in many cases are indicative of chromosomal rearrangements.

In this context, a cytotaxonomic characterization of the 7 species and 3 subspecies of Neotropical snakes of family Boidae was conducted in this study using classical and molecular chromosomal markers. Chromosomal characteristics are described for each analysed taxon to provide a new perspective on the current cytotaxonomy of these Neotropical snakes.

## Materials and Methods

Chromosomal preparations were obtained through *in vitro* culture of blood [26] from 49 animals (Table 1). All steps of this study were performed in accordance with the guidelines established by the Animal Ethics Committee of the National Institute of Amazonian Research (Protocol 013/2014).

**Table 1:** Species/subspecies studied, location, gender and number of analysed individuals.

Species	Location	Gender	No. of individuals
*Boa constrictor constrictor*	Centro de Triagem de Animais Silvestres—Manaus; Parque Estadual Rio Negro Setor Sul (Triage Centre of Wild Animals—Manaus; Rio Negro State Park South Sector); BR 174/Manaus—AM	7♂ 8 ♀	16
	Faculdades Integradas do Tapajós/Floresta Nacional do Tapajós (Integrated Colleges Tapajós/Tapajós National Forest)–PA	1 ♀	
*Boa constrictor amarali*	Fundação Zoobotânica de Belo Horizonte (Belo Horizonte Zoo-Botanical Foundation) / Belo Horizonte—MG	4 ♀ 6 ♂	10
*Boa constrictor occidentalis*	Jibóias Brasil (Boas Brazil) / Belo Horizonte—MG	1 ♂	1
*Corallus hortulanus*	Fundação Zoobotânica de Belo Horizonte (Belo Horizonte Zoo-Botanical Foundation) / Belo Horizonte—MG	1 ♂ 1 ♀	2
*Eunectes murinus*	Centro de Instrução de Guerra na Selva (Jungle Warfare Training Centre) / Manaus—AM	3 ♀ 2 ♂	10
	Lago Catalão (Catalão Lake) / Manaus—AM	1♀	
	Novo Aripuanã –AM	1 ♀	
	Área de Proteção Ambiental Acariquara (Acariquara Environmental Protection Area) / Manaus—AM	1 ♂	
	Floresta Nacional do Tapajós (Tapajós National Forest)–PA	1 ♂	
	Fundação Zoobotânica de Belo Horizonte (Belo Horizonte Zoo-Botanical Foundation) / Belo Horizonte—MG	1 ♂	
*Eunectes notaeus*	Fundação Zoobotânica de Belo Horizonte (Belo Horizonte Zoo-Botanical Foundation) / Belo Horizonte—MG	1 ♀	1
*Epicrates cenchria*	Jibóias Brasil (Boas Brazil) / Belo Horizonte—MG	1 ♀ 1 ♂	2
*Epicrates assisi*	Jibóias Brasil (Boas Brazil) / Belo Horizonte—MG	3 ♀ 1 ♂	4
*Epicrates crassus*	Fundação Zoobotânica de Belo Horizonte (Belo Horizonte Zoo-Botanical Foundation) / Belo Horizonte—MG; Jibóias Brasil (Boas Brazil) / Belo Horizonte—MG	2♀ 1 ♂	3

Analysis of the constitutive heterochromatin was performed following the protocol described by Sumner [27] with some modifications. Samples were treated with 0.2 N hydrochloric acid (HCl) at 45°C for 2 minutes and with 5% alkali barium hydroxide (Ba(OH)_2_) at 45°C for 1 minute. Then, the samples were treated with a saline sodium citrate solution (2X SSC) at 60°C for 30 minutes. Finally, the samples were stained with 5% Giemsa for 5 minutes. The detection of ribosomal active sites was performed according to the protocol described by Howell and Black [28] with some modifications. The samples were pretreated with 0.2 N HCl for 5 minutes at room temperature and then stained with AgNO_3_ for 5 minutes at 45°C. FISH was conducted according to the protocol described by Pinkel et al. [29] with modifications using 77% stringency (2.5 ng/μl of DNA, 50% deionized formamide, 10% dextran sulfate, and 2X SSC at 37°C for 24 h). The slides were counter-stained with 4',6-diamidino-2-phenylindole (DAPI). We used homologous probes for 18S rDNA [30] and telomeric probes [31] labelled with digoxigenin-11-dUTP (Dig-Nick Translation Mix, Roche).

## Results

### Karyotype with conventional staining

**Fig 1:**
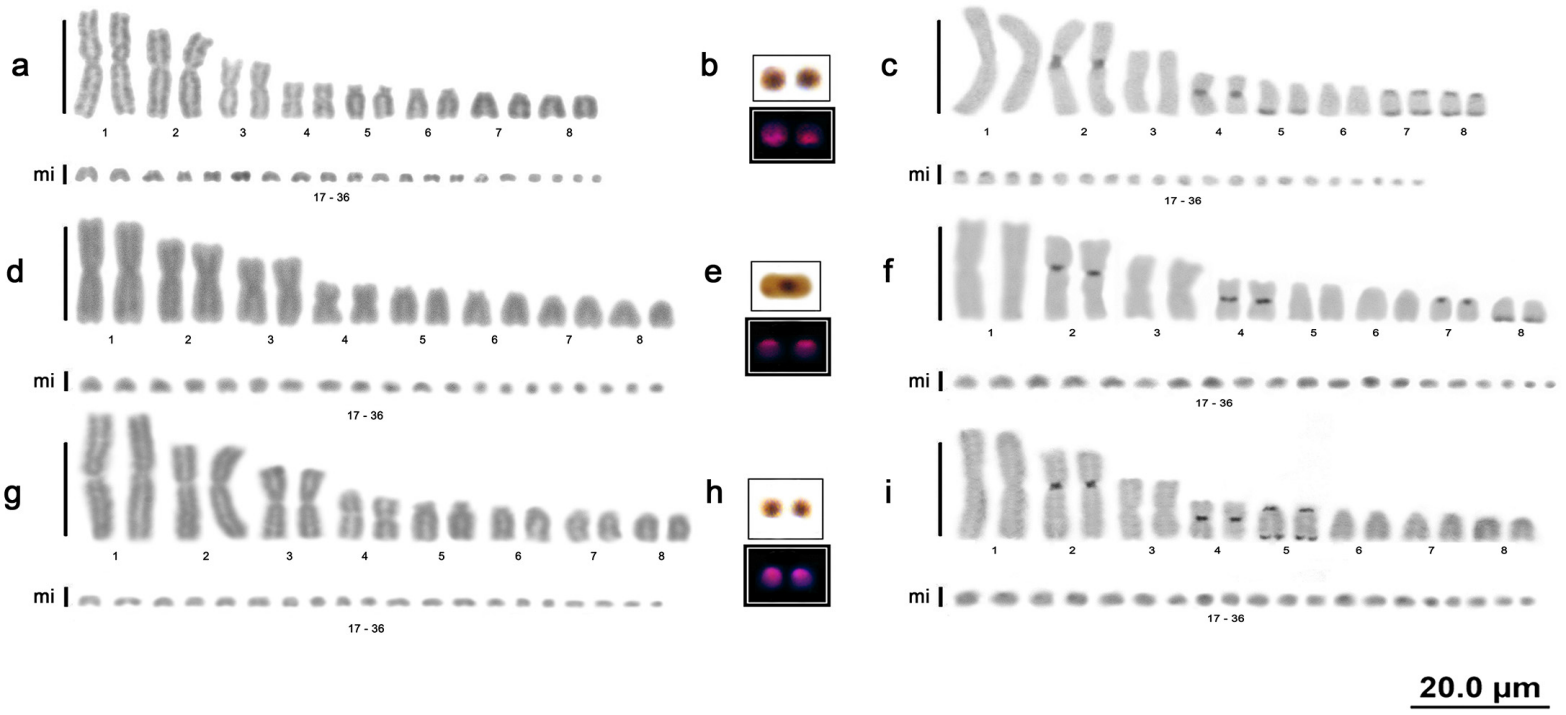
Boa karyotypes. Karyotype of *Boa constrictor constrictor* with conventional staining (a), nucleolar organizer regions and location of the 18S rDNA (b), and C-banding (c); karyotype of *Boa constrictor amarali* with conventional staining (d), nucleolar organizer regions and location of the 18S rDNA (e), and C-banding (f); karyotype of *Boa constrictor occidentalis* with conventional staining (g), nucleolar organizer regions and location of the 18S rDNA (h), and C-banding (i).

All of the *B*. *c*. *constrictor*, *B*. *c*. *amarali* and *B*. *c*. *occidentalis* samples had 2n = 36 chromosomes, the karyotype formula 6m+2sm+4st+4a+20mi and a number of arms (NF) equal to 48. No sex chromosome heteromorphisms or differences among subspecies were observed. Additionally, no differences were detected among animals from different locations (Fig 1a, 1d and 1g).

**Fig 2:**
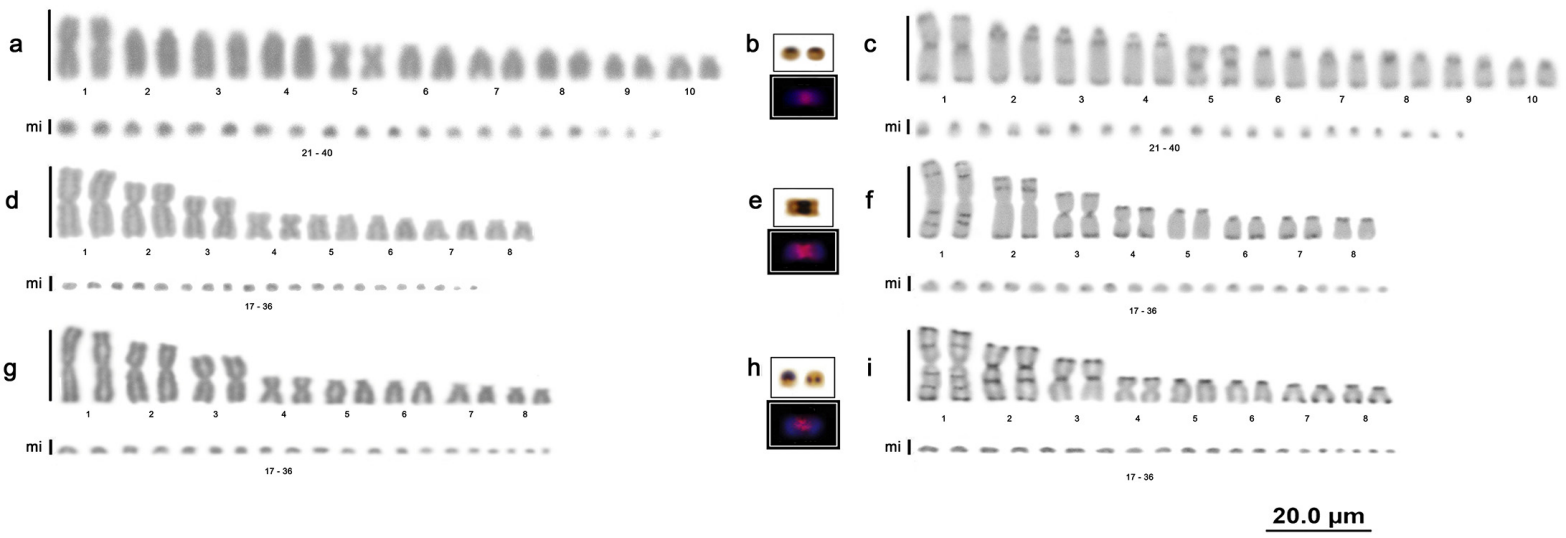
Amazon tree boa and anaconda karyotypes. Karyotype of *Corallus hortulanus* with conventional staining (a), nucleolar organizer regions and location of the 18S rDNA (b), and C-banding (c); karyotype of *Eunectes murinus* with conventional staining (d), nucleolar organizer regions and location of the 18S rDNA (e), and C-banding (f); karyotype of *Eunectes notaeus* with conventional staining (g), nucleolar organizer regions and location of the 18S rDNA (h), and C-banding (i).

The C. *hortulanus* samples had 2n = 40 chromosomes, the karyotype formula 4m+16st+20mi and an NF equal to 60, with no sex chromosome heteromorphisms (Fig 2a).

The *E*. *murinus* and *E*. *notaeus* samples had 2n = 36 chromosomes, the karyotype formula 6m+2sm+8st+20mi and an NF equal to 52, with no sex chromosome heteromorphisms or differences observed among animals from different locations (Fig 2d and 2g).

**Fig 3:**
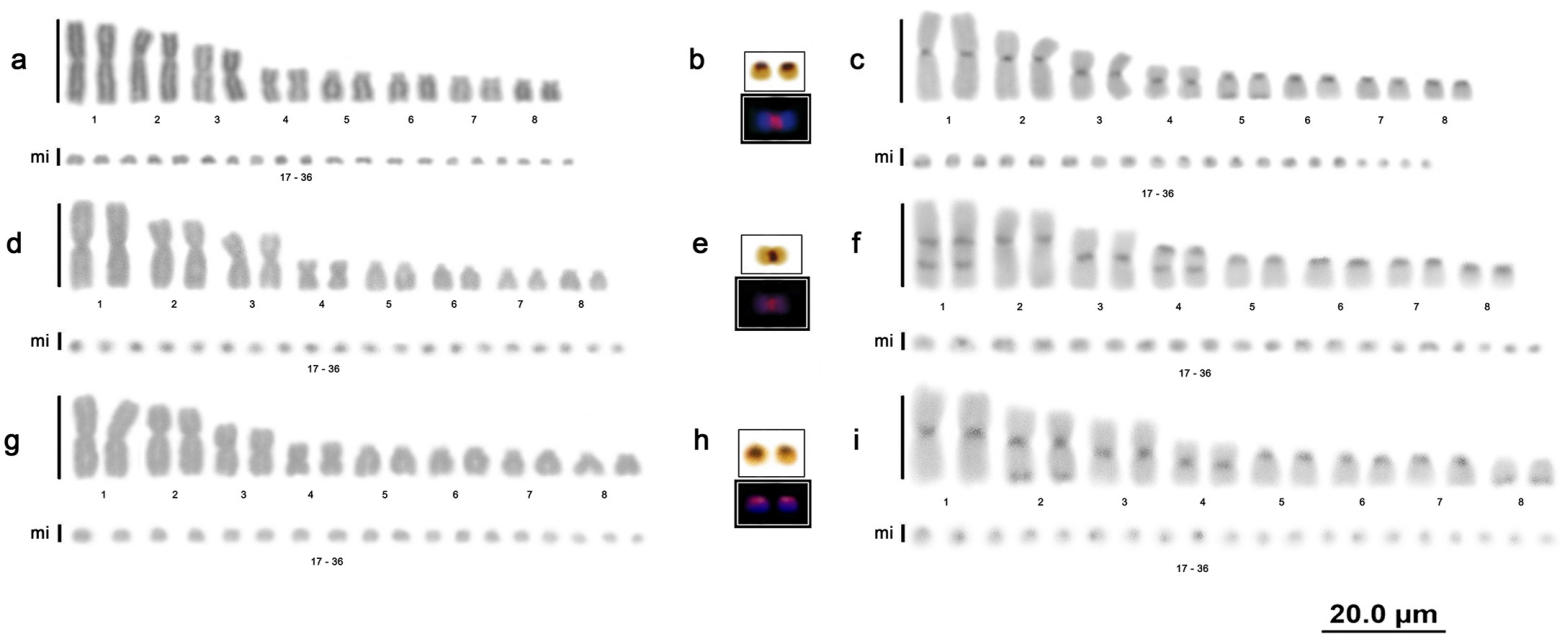
Rainbow boa karyotypes. Karyotype of *Epicrates cenchria* with conventional staining (a), nucleolar organizer regions and location of the 18S rDNA (b), and C-banding (c); karyotype of *Epicrates assisi* by conventional staining (d), nucleolar organizer regions and location of the 18S rDNA (e), and C-banding (f); karyotype of *Epicrates crassus* by conventional staining (g), nucleolar organizer regions and location of the 18S rDNA (h), and C-banding (i).

The E. *cenchria*, *E*. *assisi* and *E*. *crassus* samples had 2n = 36 chromosomes, the karyotype formula 6m+2sm+8st+20mi and an NF equal to 52, with no sex chromosome heteromorphisms (Fig 3a, 3d and 3g).

### Nucleolar organizer region (NOR) and 18S rDNA

All Boidae species analysed in this study (*B*. *c*. *constrictor*, *B*. *c*. *amarali*, *B*. *c*. *occidentalis*, *C*. *hortulanus*, *E*. *murinus*, *E*. *notaeus*, *E*. *cenchria*, *E*. *assisi* and *E*. *crassus*) presented a simple NOR on 1 pair of microchromosomes, which was confirmed by mapping of the 18S rDNA sequences (highlighted in Figs 1b, 1e, 1h, 2b, 2e, 2h and 3b, 3e and 3h).

### Constitutive heterochromatin

The distribution of C bands was primarily evident in the macrochromosomes of all individuals analysed, and a distinct pattern was observed for each species/subspecies analysed. The microchromosomes showed diffuse markings. No differences in the C-banding patterns were found among samples from different locations or between males and females.

The subspecies *B*. *c*. *constrictor* presented heterochromatic blocks in pairs 2, 4, 5, 7 and 8 (Fig 1c); *B*. *c*. *amarali* presented heterochromatic blocks in pairs 2, 4, 7 and 8 (Fig 1f); and *B*. *c*. *occidentalis* presented heterochromatic blocks in pairs 2, 4 and 5 (Fig 1i).

In *C*. *hortulanus*, heterochromatic blocks were evident in all macrochromosomes (1–20), with bitelomeric, interstitial and centromeric markings (Fig 2c).

In the anacondas (*E*. *murinus* and *E*. *notaeus*), heterochromatic blocks were also evident in all macrochromosomes (1–16), with bitelomeric, interstitial and centromeric markings (Fig 2f). In both species, the chromosome 1 pair showed high similarity, with an interstitial heterochromatic block on the short arm, 2 interstitial blocks on the long arm and bitelomeric markings; however, variations in the distribution and quantity of these heterochromatic blocks were very evident between the 2 species (Fig 2i).

In the rainbow boas (*E*. *cenchria*, *E*. *assisi* and *E*. *crassus*), we also found evident heterochromatic blocks in all macrochromosomes (1–16), with bitelomeric, interstitial and centromeric markings (Fig 3c).

### Telomeric sequences

Chromosomal mapping of the telomeric sequences revealed the presence of terminal markings on all chromosomes of all taxa analysed as well as the presence of ITSs. The exception was *E*. *notaeus*, in which no trace of an ITS was detected.

**Fig 4:**
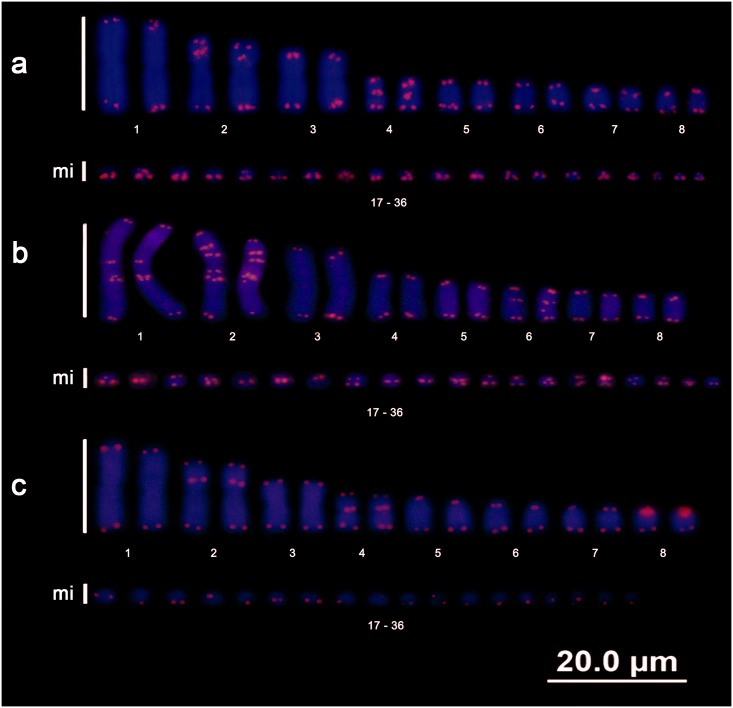
Boa telomeres. Karyotypes of *Boa constrictor constrictor* (a), *Boa constrictor amarali* (b), and *Boa constrictor occidentalis* (c) showing the distribution patterns of telomeric sequences.

**Fig 5:**
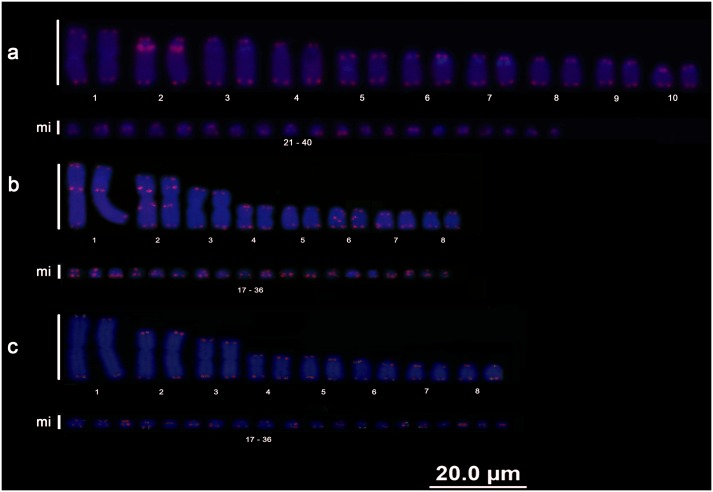
Amazon tree boa and anaconda telomeres. Karyotypes of *Corallus hortulanus* (a), *Eunectes murinus* (b), and *Eunectes notaeus* (c), showing the distribution patterns of the telomeric sequences.

**Fig 6:**
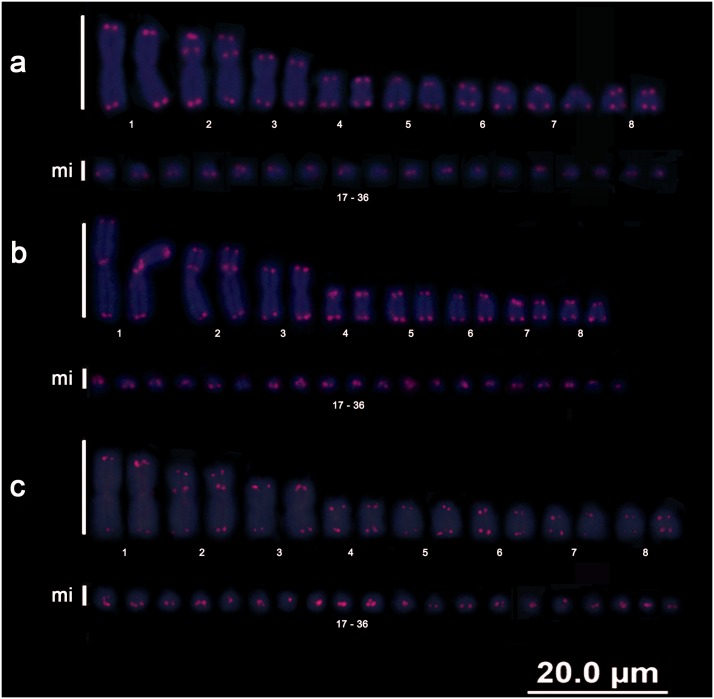
Rainbow boa telomeres. Karyotypes of *Epicrates cenchria* (a), *Epicrates assisi* (b), and *Epicrates crassus* (c) showing the distribution patterns of telomeric sequences.

*B*. *c*. *constrictor* presented ITSs in chromosome pairs 2 and 4 (Fig 4a), *B*. *c*. *amarali* in pairs 1, 2 and 6 (Fig 4b), and *B*. *c*. *occidentalis* in pairs 2, 4 and 8 (Fig 4c). *C*. *hortulanus* presented ITSs in pair 2 (Fig 5a), whereas *E*. *murinus* presented ITSs in pairs 1, 2 and 6 (Fig 5b). Furthermore, *E*. *cenchria* presented ITSs only in pair 2 in the short arm (Fig 6a), whereas *E*. *assisi* presented ITSs in pairs 1 and 2 (Fig 6b). *E*. *crassus* also presented ITSs only in pair 2 (Fig 6c).

## Discussion

### Cytotaxonomy and karyotypic organization in Neotropical boid snakes

Cytogenetic studies have revealed interesting and contrasting information about reptile evolution, such as the existence of bimodal karyotypes with macrochromosomes and microchromosomes or only macrochromosomes and heteromorphic and homomorphic sex chromosomes [2, 22].

Macrochromosomes and microchromosomes are often present in reptiles [4, 32, 33], but they are not exclusive to this group. They can also be found in other vertebrates with varying sizes and quantities [34, 35]. Asymmetric karyotypes with macrochromosomes and microchromosomes are common in snakes. The exceptions are some species in derived lineages, such as those in the genera *Thamnodynastes*, *Hydrodynastes*, *Erythrolamprus* and *Helicops* of the families Dipsadidae and Colubridae, which have karyotypes composed only of macrochromosomes ([2], unpublished data).

Although snakes of the genus *Boa* have very similar karyotype complements with a diploid number equal to 36 chromosomes, including 16 macrochromosomes and 20 microchromosomes ([1, 36], present study), differences in the karyotype formulas were observed among the samples of the 3 subspecies of *B*. *constrictor* analysed here and the samples of *B*. *c*. *amarali* and *B*. *c*. *constrictor* analysed by Beçak et al. [36]. The differences between studies are due to differences in the separation of the submetacentric and acrocentric chromosomes; whereas Beçak et al. [36] reported the formula as 6m+2sm+8a+20mi, the present study reported it as 6m+2sm+4st+4a+20mi. This difference is related to variations in both the degree of chromosomal compaction and the chromosome preparation quality.

**Fig 7:**
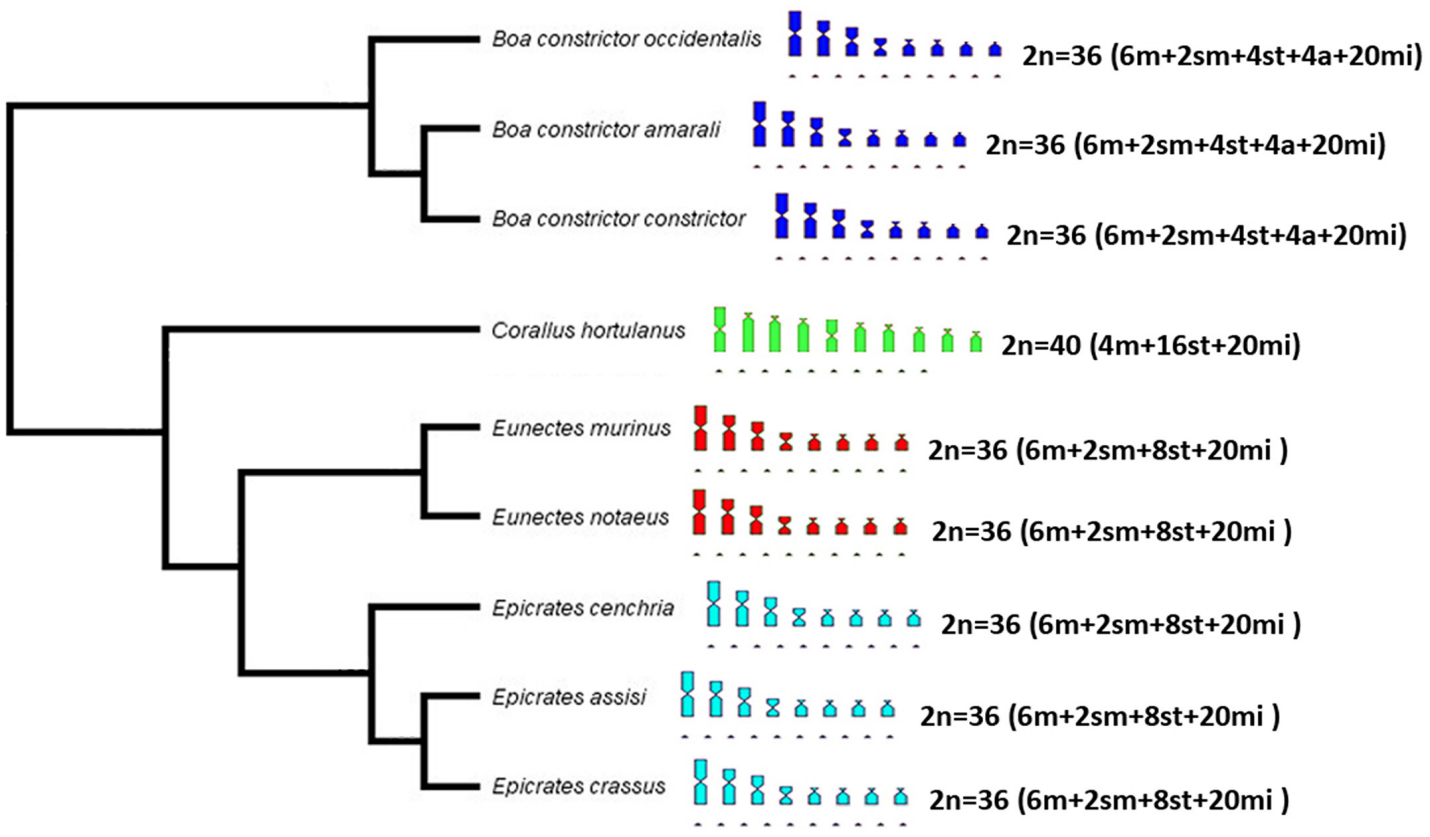
Phylogenetic relationships of the Boidae family (genera *Boa*, *Corallus*, *Eunectes* and *Epicrates*), with representation of the diploid numbers and karyotype formulas in ideograms (topology follows [37]).

Regarding the *Eunectes* and *Epicrates* snakes, the 2 species of anacondas and 3 species of rainbow boas have very similar karyotypes, as shown by conventional Giemsa staining. This finding is supported by the phylogeny proposed by Reynolds et al. [37], who indicated that these species were sister groups (Fig 7). The species of *Eunectes* and *Epicrates* only differ from the 3 subspecies of *Boa* due to the presence of 2 additional pairs of subtelocentric macrochromosomes, which are acrocentric in the *Boa* subspecies. In contrast, *C*. *hortulanus* has a completely different karyotype according to both the diploid number and karyotype formula, with fewer metacentric and submetacentric chromosomes and more subtelocentric chromosomes, which can be considered an apomorphy for the clade. This karyotypic composition more closely resembles the compositions of the species of the genera *Eunectes* and *Epicrates* than the compositions of the subspecies of *B*. *constrictor*, which is also evident in the phylogenetic relationships of the group (Fig 7).

The snakes *C*. *hortulanus* and *C*. *caninus* have the highest diploid numbers in the Boidae family, with 40 and 44 chromosomes, respectively ([1], this study). The apomorphy detected in *Corallus* (2n = 40 in this study and 2n = 44 in [1]) may have originated from fissions in the macrochromosomes from the common ancestor among *Corallus*, *Eunectes* and *Epicrates*.

To date, the karyotypes of only 2 species of the *Corallus* genus have been described: *C*. *caninus*, with a karyotype consisting of 44 basically acrocentric chromosomes [1], and another species previously known as *Corallus enhydris cookii* from the island of Grenada in the West Indies, with a karyotype of 2n = 40 chromosomes, which was observed in a female snake [38]. The latter species (*C*. *e*. *cookii*) was synonymized with *C*. *hortulanus* years later, which led us to believe that the specimen whose karyotype was described in the work by Gorman and Gress [38] was actually a specimen of *C*. *grenadensis* endemic to the island of Grenada; this species was maintained as a synonym of *C*. *hortulanus* for a long period of time. Thus, using molecular techniques, Coulston et al. [39] challenged the validity of *C*. *grenadensis*. However, Reynolds et al. [37] and Pyron et al. [10] provided strong support for the validity of this species. Furthermore, Gorman and Gress [38] reported a karyotype of 2n = 40, 4m+16a+20mi, and NF = 44 ♀ for *C*. *grenadensis*, which was significantly different from the karyotype of *C*. *hortulanus* described in the present work in terms of the fundamental number and karyotype formula (2n = 40, 6m+16st+18mi, and NF = 62 ♀). Therefore, we consider that this is the first karyotypic description of *C*. *hortulanus* from South America.

### Variations in the ribosomal regions and 18S rDNA sequences in snakes

The number of nucleolar markings and their locations on a pair of microchromosomes demonstrated by Ag-NOR staining and 18S rDNA sequences can be considered conserved features among snakes of the basal lineage [23] and plesiomorphic features of several families of snakes with 2n = 36 chromosomes, including 16 macrochromosomes and 20 microchromosomes [23, 40, 41]. In contrast, there is great variation in the locations of these markings among some snakes of derived lineages, which may be present on macrochromosomes, microchromosomes and even sex chromosomes [23, 42, 43, 44]. These variations in the locations of rDNA sites have been well documented for the species *Bothrops neuwiedi* [45], in which rDNA is present in both the microchromosomes and macrochromosomes in different populations. This characteristic supports the hypothesis proposed by Camper and Hanks [23], who suggested that translocation rearrangements occurred between the macrochromosomes and microchromosomes, which in this case changed the location of the nucleolar region while the number of microchromosomes remained the same.

These variations were also reported by Porter et al. [40], who identified variability in the locations of ribosomal sites using FISH with 28S rDNA probes in several snake species from different families. *Thamnophis marcianus* (Natricidae) and *Coluber flagellum* (Colubridae) were found to have simple markings on macrochromosomes, whereas *Crotalus viridis* (Viperidae) presented multiple markings on 2 pairs of microchromosomes. A similar pattern was evident in *Bothrocophias hyoprora* (Viperidae) from the Amazon region, which was found to have multiple markings on 2 pairs of microchromosomes based on staining with AgNO_3_ [46]. Additionally, staining with AgNO_3_ together with 18S rDNA mapping using homologous probes allowed the identification of simple markings on a single pair of macrochromosomes in *Spilotes sulphureus* (Colubridae). No association of rDNA with the sex chromosomes of this species was detected (unpublished data). These characteristics (NOR, 18S and 28S rDNA) along with simple markings of a pair of macrochromosomes and multiple markings of both macrochromosomes and microchromosomes are typical peculiarities of snakes belonging to intermediate and derived lineages.

### Specificity of the C-banding patterns in Neotropical boid snakes

The distribution pattern of constitutive heterochromatin was specific for each species/subspecies of *Boa*, *Corallus*, *Eunectes* and *Epicrates*, making this pattern an excellent cytotaxonomic marker for differentiating among all of the Boidae snakes analysed here. There is wide variation in the C-banding patterns among the snakes of the basal lineages, especially compared with the genera that constitute the Boidae family and the Booidea superfamily. The subspecies of *Boa* have small amounts of constitutive heterochromatin relative to the sizes of their macrochromosomes (present study), whereas *Sanzinia* (Sanziniidae) possesses large heterochromatic blocks [4].

For the *Boa* subspecies, the blocks in pairs 2 and 4 and the absence of heterochromatin in pairs 1, 3 and 6 suggest the existence of a high level of similarity among these taxa. However, the variation present in the other macrochromosomes (pairs 5, 7 and 8) supports the phylogeny proposed by Reynolds et al. [37], who suggested that the subspecies analysed in the present study indeed belonged to distinct clades (Fig 7). Hynková et al. [6] suggested that the subspecies *B*. *c*. *imperator* should be elevated to the category of species due to differences from other *B*. *constrictor* subspecies. There are still no cytogenetic data for this species; these data would represent a major breakthrough in understanding the current taxonomy of the genus. However, the mapping of telomeric sequences and the constitutive heterochromatin patterns (which were subspecies-specific) suggested that *B*. *c*. *constrictor*, *B*. *c*. *amarali* and *B*. *c*. *occidentalis* could be elevated to the rank of full species.

In *Eunectes*, pair 1 is apparently similar and conserved between the 2 species analysed; these snakes possessed the greatest amounts of constitutive heterochromatin compared with *Boa*, *Epicrates* and *Corallus*. Although notable differences were observed between the heterochromatin patterns in *Eunectes* and *Epicrates*, these patterns were clearly much more similar to one another than to the heterochromatin patterns observed in the *B*. *constrictor* subspecies in terms of both the number of blocks and their distribution among the macrochromosomes. In *C*. *hortulanus*, the heterochromatin pattern was also more similar to the patterns in *Eunectes* and *Epicrates* than to the patterns in the *B*. *constrictor* subspecies, especially with regard to the karyotype formulas and the presence of very evident heterochromatic blocks in pair 1 in all species of *Corallus*, *Eunectes* and *Epicrates*, which were non-existent in the 3 *Boa* subspecies.

No differences related to differentiated sex chromosomes were found in *Boa*, *Corallus*, *Eunectes* or *Epicrates* (present study), which is a common characteristic of the basal taxa of snakes, such as those of the family Boidae. Sex chromosome heteromorphisms are observed more often in derived taxa [2, 3, 21, 22], although there are some exceptions, such as *Acrantophis dumerili* (Sanziniidae), which exhibits sex chromosome differentiation [4]; this is the only case of sex chromosome heteromorphism recorded in Henophidia to date. However, *A*. *dumerili* may not be the only exception of sex chromosome heteromorphism in this group (unpublished data).

### Telomere-specific patterns and chromosomal rearrangements in Neotropical boid snakes

To date, no mapping of telomeric sequences or traces of ITSs indicative of chromosomal rearrangements have been identified in representative species of the Boidae family.

In our study, we demonstrated patterns that distinguished each species/subspecies. Additionally, we performed chromosomal mapping of telomeric sequences and revealed the notable occurrence of various ITSs, which were evident in the macrochromosomes of the 3 subspecies of *B*. *constrictor*, *C*. *hortulanus*, and *E*. *murinus* and the 3 species of *Epicrates*; these ITSs may be indicative of chromosomal rearrangements. These findings suggest that ITSs may not necessarily randomly occur in Boidae snakes but may represent an independent evolutionary history for each genus.

The ITSs present among the various orders of vertebrates have different origins [47–51]. For example, ITSs may arise due to the activity of telomerase in repairing chromosomal breaks by adding telomeric sequences to non-terminal regions [52, 53] resulting from fusions, fissions, inversions or even duplications of these sequences [47,54]. For example, duplications appear to have occurred in the last macrochromosome pair of *B*. *c*. *occidentalis* and in pair 2 of *C*. *hortulanus*. The ITS present in pair 2 (st) of *C*. *hortulanus* may be indicative of an inversion, followed by the duplication of (TTAGGG)n fragments. This ITS would be equivalent to the pairs of submetacentric macrochromosomes maintained in *Boa*, *Eunectes* and *Epicrates* because the ITS was associated with a heterochromatic region, and there was an ITS at an interstitial position on the short arm of pair 2 (sm) in all analysed boid snakes, with the exception of *E*. *notaeus*.

In some groups, there is an apparent distribution pattern of telomeric motifs, which may indicate a common evolutionary history between sister clades, such as *Eunectes* and *Epicrates*. The mapping of telomeric sequences in *E*. *cenchria* and *E*. *crassus* revealed that these species had very similar compositions, including identical ITS patterns. Interestingly, despite the similar distribution patterns of telomeric sequences, *E*. *cenchria* and *E*. *crassus* appear to exhibit patterns that are more similar to those of *Eunectes* than to those of the *Boa* subspecies, especially because *E*. *notaeus* does not possess ITSs.

Notably, the presence of ITSs is common in squamates [25]. These sites may be considered common even in basal clades, such as the family Boidae (this study), highlighting the role of the dynamics of chromosomal rearrangements in the diversity of this group. However, we believe that not all ITSs found in the snakes analysed here are necessarily indicative of chromosomal rearrangements because not all of them are associated with constitutive heterochromatin, although it is highly probable that they are associated with transposable elements [55, 56] or satellite DNA [57]. In contrast, no correlation was detected between the ITSs of the species *Eunectes murinus* and the non-long terminal repeat (LTR) retrotransposon Rex6 (unpublished data). Although not all of the ITSs located in pair 2 (sm) of *Boa*, *Eunectes* and *Epicrates* are associated with heterochromatin, it is very likely that these ITSs are indicative of chromosomal rearrangements and that they are a result of recent events.

Further study is needed to determine whether other repetitive elements are associated with the ITSs found in these snakes and to uncover the real reason why some species are susceptible to the accumulation of these sequences in their chromosomes.

## Conclusions

The data obtained in this study increase our understanding of the cytotaxonomy of Neotropical Boidae snakes. Specific chromosomal characteristics were identified for each taxa, revealing that the karyotypes of these snakes were not as conserved as previously thought. Our study is the first to identify ITSs in the Boidae family and superfamily Booidea as well as in Henophidia snakes. These findings suggest that chromosomal rearrangements have contributed to the diversification of the group. We also conclude that ITSs are common in basal and derived lineages within the suborder Serpentes. Thus, our study significantly contributes to current knowledge regarding the taxonomy and karyotype organization of the group.
